# Supplementary material for: WordCommentsAnalyzer: A windows software tool for qualitative research
Source: F1000Res. 2018 Sep 4;7:536. Originally published 2018 May 3. [Version 2] doi: 10.12688/f1000research.14819.2 (PMC6137411; doi:10.12688/f1000research.14819.2)
Supplement: Supplementary file 3 [file f1000research-7-17614-s0002.tgz › b2a79496-4ada-47c4-9682-a99ce867836d.zip!/Eight Iranian Health Journals/Journal of Health System Research _44.docx]

© Journal of Health System Research . This work is licensed under CC BY-NC 4.0 (https://creativecommons.org/licenses/by-nc/4.0/).

Volume & Issue: دوره 7، شماره 1؛ 1390

Title: ارتباط دانسيته انرژي رژيم غذايي با چاقي

http://hsr.mui.ac.ir/index.php/jhsr/article/view/125

مقدمه: مطالعات زيادي در زمينه ارتباط دانسيته انرژي رژيم غذايي با چاقي صورت گرفته، اما مطالعه‌اي در اين رابطه در ايران صورت نگرفته است. بنابراين مطالعه حاضر با هدف بررسي ارتباط دانسيته انرژي رژيم غذايي با چاقي در بين دانشجويان دختر دانشکده بهداشت دانشگاه علوم پزشکي اصفهان صورت گرفته است. روش‌ها: در اين مطالعه مقطعي 90 نفر از دانشجويان دختر دانشکده بهداشت به روش خوشه‌اي انتخاب شدند. دريافت‌هاي غذايي آن‌ها به وسيله يک پرسشنامه نيمه کمي بسامد خوراک 168 آيتمي ارزيابي گرديد. قد، وزن و دور کمر نيز اندازه‌گيري شد. منظور از چاقي عمومي در اين مطالعه نمايه توده بدني بزرگ‌تر از 25 و منظور از چاقي شکمي داشتن دور کمر بيشتر از 80 سانتي‌متر است. یافته‌ها: در افراد مورد مطالعه ميانگين نمايه توده بدني، دانسيته انرژي رژيم غذايي و کل انرژي دريافتي به ترتيب  1/21 کیلوگرم بر مترمربع، 0/87 کیلوکالری بر گرم و 2122 کیلوکالری بود. شيوع چاقي عمومي 9 درصد و شيوع چاقي شکمي 9/8 درصد بود. رابطه معکوس و معني‌داري بين دانسيته انرژي رژيم غذايي و نمايه توده بدني (03/0=P و 9/4-=β ) حتي پس از تعديل عوامل مخدوش‌گر (04/0=P و 4/5=β) مشاهده شد. اما رابطه معني‌داري بين دور کمر و دانسيته انرژي رژيم غذايي وجود نداشت. نتيجه‌گيري: با وجود نبودن تفاوت معني‌دار در شيوع چاقي در بين رده‌هاي دانسيته انرژي، به نظر مي‌رسد نمايه توده بدني با دانسيته انرژي رابطه داشته باشد.

Title: بررسی اثرات مصرف لبنیات و کلسیم بر چاقی و چاقی شکمی در دختران دانشجوی دانشگاه علوم پزشکی اصفهان

http://hsr.mui.ac.ir/index.php/jhsr/article/view/120

مقدمه: امروزه چاقی به عنوان یک مشکل جهانی تلقی می‌شود. چاقی با این که زمینه‌ای از آن ژنتیکی است، اما عوامل محیطی و تغذیه‌ای، از عوامل مستعد کننده آن می‌باشند. مطالعات متعددی به ارتباط عوامل تغذیه‌ای با چاقی و چاقی شکمی اشاره کردند. از این رو در این تحقیق، به بررسی ارتباط مصرف لبنیات با شیوع چاقی و چاقی شکمی در دختران دانشجوی دانشگاه علوم پزشکی اصفهان پرداخته شد. روش‌ها: اين تحقيق بر روي 236 نفر دانشجوي دختر دانشگاه علوم پزشكي اصفهان كه در محدوده سني 30-18 سال قرار داشتند، به روش مقطعي انجام شد. دانشجویان به روش تصادفی انتخاب شدند. از افراد فرم بسامد خوراك نیمه‌کمی اعتبار سنجی‌شده گرفته شد. ارزیابی فعالیت بدنی با استفاده از ثبت روزانه فعالیت بدنی بررسی شد. یافته‌ها: نتایج این مطالعه نشان می‌دهند که شیوع چاقی 7/1 درصد و چاقی شکمی 9/0 درصد و اضافه وزن 1/8 درصد می‌باشد. میانگین BMI و دور کمر به ترتیب برابر 54/21 کیلوگرم بر متر مربع و 37/70 سانتی‌متر می‌باشد. میانگین دریافت لبنیات برابر 24/444 گرم در روز است. نتایج هیچ‌گونه ارتباط معنی‌داری میان مصرف لبنیات و یا کلسیم با وزن، دور کمر و همچنین شیوع چاقی، چاقی شکمی و اضافه وزن نشان نمی‌دهند (05/0P > ). نتیجه‌گیری: ارتباط معنی‌داری بین مصرف لبنیات و کلسیم با اضافه وزن، چاقی و چاقی شکمی در دانشجویان دختر دیده نشد. اما انجام این طرح از جهت گزارش میزان شیوع چاقی، چاقی شکمی و اضافه وزن و چگونگی وضعیت نمایه توده‌ بدنی و دور کمر حایز اهمیت می‌باشد.

Title: بررسی اثر مصرف شیر سویا بر شاخص‌های آنتروپومتریک و فشار خون در دختران مبتلا به اضافه وزن و چاقی

http://hsr.mui.ac.ir/index.php/jhsr/article/view/165

مقدمه: رژیم غذایی حاوی شیر سویا، ممکن است اثرات مفیدی بر شاخص‌های تن‌سنجی و میزان فشار خون در افراد مبتلا به اضافه وزن و چاقی داشته باشد. بنابراین ما می‌خواهیم اثرات شیر سویا را بر وزن ،دور کمر و فشار خون در دختران جوان مبتلا به اضافه وزن و چاقی بررسی کنیم. روش‌ها: این بررسی، یک کارآزمایی بالینی مقاطع و تصادفی بود که بر روی 23 دختر جوان مبتلا به اضافه وزن و چاقی انجام شد. شرکت کنندگان به طور تصادفی به 2 گروه دریافت کننده شیر سویا و گروه شیر گاو به مدت 6 هفته تقسیم شدند. بین دو دوره نیز 3 هفته دوره Wash- out قرار داشت و به هر دو گروه هم رژیم کاهش وزن داده شد. در دوره شیر سویا تنها یک لیوان شیر سویا (CC 240) جایگزین یک لیوان شیر گاو (CC 240) شد. یافته‌ها: میانگین سن بیماران 2 ± 22 سال و میانگین BMI 0/50 ± 28/1 کیلوگرم بر متر مربع بود. وزن و دور شکم به طور معنی‌دارای بعد از دوره شیر سویا در مقایسه با دوره شیر گاو تغییر نکرد. (میانگین درصد تغییرات در دوره شیر سویا برای وزن 4/0 ± 6/3- در مقابل 2/0 ± 0/1-) در دوره شیر گاو 82/0 = P دور شکم 2/0 ± 8/0- در مقابل 11/0 = P؛ 2/0 ± 1/0-، فشار خون سیستولیک به طور معنی‌داری در طی دوره شیر سویا کاهش پیدا کرد. (میانگین درصد تغییرات در دوره شیر سویا 0/9 ± 0/4- در مقابل 5/0 ± 7/1- در دوره شیر گاو؛ (05/0 > P). فشار خون دیاستولیک نیز در دوره شیر سویا کاهش پیدا کرد (1/0 ± 4/0- در مقابل 1/0 ± 4/0؛ 05/0 > P). نتیجه‌گیری‌: رژیم غذایی حاوی شیر سویا هم فشار خون سیستولیک و هم دیاستولیک را در دختران جوان مبتلا به اضافه وزن و چاقی کاهش داد. اگر چه جایگزین کردن شیر سویا در رژیم غذایی هیچ نتیجه معنی‌داری بر روی وزن و دور شکم نداشت.

Title: ارتباط مصرف میوه و سبزی با نمایه توده بدنی و دور کمر در دختران جوان دانشجو

http://hsr.mui.ac.ir/index.php/jhsr/article/view/167

مقدمه: در کشور ما شیوع چاقی به سرعت رو به گسترش است، که تغذیه از جمله موارد مرتبط با آن می‌باشد. هدف از انجام این طرح بررسی نقش میوه و سبزی در ارتباط با دور کمر و BMI (Body Mass Index) در دختران جوان دانشجو است. روش‌ها: این تحقیق بر روی 236 نفر دانشجوی دختر دانشگاه علوم پزشکی اصفهان که در رنج سنی 18 تا30 سال قرار دارند، به روش مقطعی انجام شد. دانشجویان به روش تصادفی انتخاب شدند و ارزیابی تغذیه‌ای با استفاده از فرم FFQ (Food Frequency Questionnaire) نیمه‌ کمی و ارزیابی فعالیت بدنی با استفاده از ثبت روزانه فعالیت بدنی بررسی شد. یافته‌ها: یافته‌ها حاکی از آن است که در بین دانشجویان دانشگاه علوم پزشکی اصفهان درصد شیوع چاقی 7/1، چاقی شکمی 9/0 و اضافه وزن 1/8 بوده است. همچنین این تحقیق نشان داد که میانگین نمایه توده بدنی و دور کمر به ترتیب 54/21 کیلوگرم بر متر مربع و 37/70 سانتی‌متر بوده، ارتباط معکوس معنی‌داری میان مصرف مجموع میوه و سبزی با وزن بدن (03/0 = P، 1/0- = r) و BMI (04/0 = P، 1/0- = r) و همچنین مصرف میوه با وزن بدن (01/0 = P، 1/0- = r) و BMI (01/0 = P، 1/0- = r) مشاهده گردید. ارتباطی بین مصرف میوه و سبزی با هم و به طور جداگانه با دور کمر دیده نشد (05/0 < P). نتیجه‌گیری: ارتباط معکوس معنی‌داری بین مصرف میوه و مجموع میوه و سبزی با وزن بدن و BMI در دانشجویان دختر دانشگاه علوم پزشکی اصفهان مشاهده گردید، اما ارتباط معنی‌داری بین مصرف میوه و سبزی با هم و به طور جداگانه با دور کمر دیده نشد.

Title: روند تغييرات 10 ساله در شاخص تأثير مجلات تغذيه در دنيا

http://hsr.mui.ac.ir/index.php/jhsr/article/view/146

مقدمه: هدف از انجام اين مطالعه بررسي شاخص تأثير و ارجاعات کل مجلات تغذيه‌اي ثبت شده در ISI در 10 سال گذشته است. روش‌ها: جهت انجام اين مطالعه گذشته‌نگر، داده‌هاي مورد نياز مربوط به شاخص تأثیر و تعداد ارجاعات مقالات درج شده در مجلات تغذيه و رژيم درماني ثبت شده در ISI در طی 10 سال گذشته جمع‌آوري شده است. در اين مطالعه 5 نمونه از مجلات دسته تغذيه و رژيم درماني ISI که از شاخص تأثیر بالايي برخوردارند (AJCN، JN، IJO، ARN و PLR) مورد آناليز قرار گرفته‌اند. یافته‌ها: 5 مجله انتخابي و مورد بررسي در اين مطالعه در 10 سال گذشته (از سال 1999 تا سال 2008) جزء 10 مجله اول ثبت شده در ISI در دسته مجلات تغذيه و رژيم درماني مي‌باشند. بيشتر مجلات انتخابي از روند صعودي شاخص تأثیر و البته همراه با نوساناتي در برخي از موارد در اين دوره 10 ساله برخوردار بوده‌اند. مجله Am J Clin Nutr به طور ثابتي از بالاترين ميزان استنادات کل برخوردار بود. نتیجه‌گیری: شاخص تأثیر معياري مناسب جهت مقايسه و بررسي کيفيت مجلات تغذيه‌اي و البته بررسي عملکرد پژوهشي محققين در مجلات مربوط به اين رشته است.

Title: بررسی اثر رژیم غذایی غنی از روغن زیتون بر سطح قند و لیپیدهای پلاسما در زنان دارای اضافه وزن

http://hsr.mui.ac.ir/index.php/jhsr/article/view/143

مقدمه: رژیم غذایی غنی از روغن زیتون به عنوان یک منبع اسیدهای چرب تک غیر اشباع، از طریق کاهش شاخص‌های لیپیدی پلاسما در کاهش بیماری‌های قلبی- عروقی نقش دارد. هدف از انجام این مطالعه بررسی اثر رژیم غذایی غنی از روغن زیتون بر سطح پلاسمایی شاخص‌های قند و لیپید پلاسما در زنان دارای اضافه وزن می‌باشد. روش‌ها: این مطالعه به صورت یک کارآزمایی بالینی متقاطع طراحی شد که درآن 20 زن سالم دارای اضافه وزن در محدوده سنی 50-20 سال و محدوده 29/9-25 = BMI، با روش تخصیص تصادفی به دو گروه تقسیم شدند. یک گروه رژیم غذایی معمولی محدود از انرژی (51 درصد کربوهیدرات، 15 درصد پروتئین، 34 درصد چربی کل، 16 درصد SFA، 8 درصد MUFA و 10 درصد PUFA) وگروه دیگر رژیم محدود از انرژی و غنی از روغن زیتون (ترکیب درشت مغذی‌ها شبیه رژیم معمولی ولی با 8 درصد SFA، 16 درصد MUFA و 10 درصد PUFA) دریافت کردند. طول مدت مداخله در هر فاز 6 هفته و بین دو فاز نیز 2 هفته wash-out بود. سطوح پلاسمایی قند، کلسترول، تری گلیسیرید، LDL و HDL در حالت ناشتا در ابتدا و انتهای هر دو فاز اندازه‌گیری شد. تغییرات شاخص‌های ذکر شده در طول هر فاز مطالعه محاسبه و با استفاده از آزمون  t‌مزدوج مقایسه گردید. یافته‌ها: سطوح پلاسمایی قند، کلسترول، تری گلیسیرید، LDL و HDL در حالت ناشتا در طول هر دو نوع رژیم غذایی کاهش یافت و میزان کاهش آن در رژیم غذایی غنی از روغن زیتون در مقایسه با رژیم غذایی معمولی اندکی بیشتر بود، ولی به حد معنی‌داری نرسیده بود. نتیجه‌گیری: رژیم غذایی غنی از روغن زیتون در مقایسه با رژیم معمولی غلظت پلاسمایی قند، کلسترول، تری گلیسیرید، LDL و HDL ناشتا را بیشتر کاهش داد، اما این اختلاف از نظر آماری معنی‌دار نبود.

Title: بررسی وضعیت دریافت انرژی از درشت مغذی‌‌ها و ارتباط آن با شاخص‌های آنتروپومتری در جانبازان اصفهان

http://hsr.mui.ac.ir/index.php/jhsr/article/view/148

مقدمه: مطالعات گوناگون نشان دادند که دریافت درشت مغذی‌ها در سبد غذایی جانبازان جنگ تحمیلی کمتر از مقادیر توصیه شده می‌باشد. در نتیجه جانبازان در معرض خطر عدم تعادل تغذیه‌ای قرار دارند. هدف از انجام این طرح ارزیابی وضعیت دریافت درشت مغذی‌ها و ترکیب بدن جانبازان شهر اصفهان بود. روش‌ها: این تحقیق یک مطالعه توصیفی- تحلیلی از نوع مقطعی بود. افراد مورد بررسی 106 نفر و جمعیت مورد مطالعه جانبازان با درصد جانبازی بالای 10 درصد شهر اصفهان بودند. جمع‌آوری اطلاعات با استفاده از پرسش‌نامه‌های فرم اطلاعات عمومی و یادآمد 2 روزه خوراک انجام شد. بررسی داده‌‌ها با نرم‌افزار تغذیه‌ای 4N و نرم‌افزار آماری 10SPSS انجام شد. یافته‌ها: در این طرح به بررسی میانگین دریافت درشت مغذی‌‌های غذایی و شاخص‌های تن سنجی در افراد جانباز پرداخته شد. همچنین مقایسه میانگین دریافت درشت مغذی با مقادیر توصیه شده (RDA) و مقایسه میانگین شاخص‌های تن سنجی با مقادیر استاندارد، انجام شد. ارتباط بین مقدار دریافت درشت مغذی‌ها با شاخص‌‌‌های تن سنجی نیز تعیین گردید. نتیجه‌گیری: با توجه به یافته‌های مطالعه، الگوی دریافت غذایی جانبازان نیاز به اصلاح دارد. دریافت برخی از درشت مغذی‌‌ها بیشتر از میزان توصیه شده بود. با توجه به یافته‌‌های مربوط به شاخص‌های تن سنجی و ترکیب بدن در این گروه که نشان می‌دهد میزان چربی کل بیش از حد نرمال است، مشخص می‌شود که فعالیت بدنی منظم نقش مهمی برای ارتقای سلامتی این افراد دارد.

Title: آنالیز آنتی‌بادی علیه هلیکوباکتر پیلوری در زنان باردار مبتلا به تهوع و استفراغ دوران بارداری

http://hsr.mui.ac.ir/index.php/jhsr/article/view/185

مقدمه: یکی از مشکلات عمده زنان باردار تهوع و استفراغ بارداری است که بین هفته‌های 7-4 بعد از LMP در 80 درصد آنان دیده می‌شود و عموماً در 20 هفتگی برطرف می‌شود. Hyper emesis gravid arum یک فرم شدید تهوع و استفراغ است که در        زنان باردار دیده می‌شود. علایم آن به صورت استفراغ پایدار، دهیدراتاسیون، کاهش بیش از 5 درصد وزن بدن، کتون اوری، اختلالات الکترولیتی از قبیل هیپوکالمی می‌باشد. عوارضی نیز چون پارگی مری، پنوموتوراکس، نوروپاتی محیطی، پره اکلامپسی، محدودیت رشد جنینی و افزایش مرگ و میر دارد. مطالعات اخیر حاکی از نقش احتمالی هلیکوباکتر پیلوری در بروز تهوع و استفراغ بارداری و هیپرامزیس گراویدوم می‌باشد. هدف از این مطالعه تعیین وضعیت تیتر آنتی‌بادی علیه هلیکوباکتر پیلوری در زنان باردار مبتلا به تهوع و استفراغ و HG در شهرستان ایلام می‌باشد. روش‌ها: این مطالعه مورد– شاهدی بر روی زنان باردار مراجعه‌کننده به درمانگاه زنان بیمارستان مصطفی خمینی و مراکز بهداشتی درمانی شهر ایلام صورت گرفت. 50 نفر در گروه مورد با تهوع و استفراغ شدید و 50 نفر در گروه شاهد (بدون تهوع و استفراغ)، به روش نمونه‌گیری غیر احتمالی سهمیه‌ای انتخاب شدند. پس از جمع‌آوری کلیه داده‌های لازم از طریق مصاحبه و تکمیل پرسش‌نامه، از نظر تیتر آنتی‌بادی IgG و IgM علیه هلیکوباکتر پیلوری، به روش ELISA مورد بررسی قرار گرفتند. کلیه اطلاعات توسط نرم‌افزار 16SPSS و آزمایش‌های مجذور خی، مان ویتنی و t-test مورد آنالیز قرار گرفتند. سطح معنی‌داری آزمون (05/0 = α) در نظر گرفته شد. یافته‌ها: میانگین تیتر IgG علیه هلیکوباکتر پیلوری در گروه مورد (9/15 ± 2/45) و در گروه شاهد (7/14 ± 8/25) به دست آمد که این اختلاف معنی‌دار می‌باشد (04/0‌ = P). همچنین میانگین تیتر IgM در گروه مورد (2/7 ± 6/19) و در گروه شاهد (9/6 ± 8/11) مشاهده گردید که این تفاوت نیز معنی‌دار است (01/0 = P). در گروه مورد بروز تهوع و استفراغ شدید ( بیش از 3 بار در روز) با افزایش تیتر IgM (عفونت حاد) معنی‌دار به دست آمد (02/0 = P). در حالی که افزایش تیتر IgG با بروز تهوع و استفراغ شدید ارتباط معنی‌داری را نشان نداد (08/0 = P). نتیجه‌گیری: هلیکوباکتر پیلوری می‌تواند به عنوان یکی از دلایل بروز تهوع و استفراغ شدید و حتی بیماری هیپرامزیس گراویداروم در زنان باردار مطرح باشد. استفاده از آزمایش‌های تشخیصی عفونت HP و در صورت لزوم درمان و ریشه‌کنی این باکتری با داروهای اختصاصی در بهبود زنان باردار، توصیه می‌گردد.

Title: بررسي وضعيت هوش هيجاني در بين دانشجويان دانشگاه‌هاي علوم پزشكي غرب كشور

http://hsr.mui.ac.ir/index.php/jhsr/article/view/187

مقدمه: هوش هيجاني يك مهارت اجتماعي است و شامل درك احساسات خود براي تصميم‌گيري مناسب و همچنين كاربرد احساسات در روابط و توانايي رهبري ساير افراد مي‌باشد. اين پژوهش به روش توصيفي و با هدف بررسي وضعيت هوش هيجاني دانشجويان دانشگاه‌هاي علوم پزشكي غرب كشور و مقايسه وضعيت هوش هيجاني در بين دانشجويان دختر و پسر اين دانشگاه‌ها صورت گرفت.  روش‌ها: جامعه آماري اين مطالعه، كليه دانشجويان دانشگاه‌هاي علوم پزشكي غرب كشور بودند كه با استفاده از روش نمونه‌گيري تصادفي- طبقه‌اي، تعداد 303 نفر از آن‌ها به عنوان نمونه آماري انتخاب گرديدند. براي جمع‌آوري داده‌ها از آزمون هوش هيجاني بار- ان استفاده شد. براي تجزيه و تحليل داده‌ها از آمار توصيفي شامل فراواني، درصد فراواني ساده، ميانه، ميانگين و انحراف استاندارد و براي سنجش فرضيات از آزمون ناپارامتريك من ويتني يو استفاده شد. یافته‌ها: نتايج اين تحقيق حاكي از اين است كه 2/83 درصد از پاسخ‌گويان در حد زياد و خيلي زيادي داراي حس همدلي و درك احساس ديگران بودند. اكثر پاسخ‌گويان (5/63 درصد) در برخورد با تغييرات و موقعيت‌ها در حد متوسطي داراي انعطاف‌پذيري بودند. همچنين ميزان برخي از مؤلفه‌هاي هوش هيجاني از قبيل حل مسأله، استقلال، خودآگاهي هيجاني، خوش‌بيني و همدلي دانشجويان دختر و پسر تفاوت معنی‌دار وجود داشت. نتیجه‌گیری: نتايج نشان مي‌دهند كه ميزان هوش هيجاني دانشجويان و به خصوص دانشجويان دختر در زمينه مؤلفه‌ها و خرده آزمون‌هاي كنترل تكانش، انعطاف‌پذيري، واقع‌گرايي، تحمل فشار رواني و خودابرازي در حد متوسط مي‌باشد. پيشنهاد مي‌شود كه در قالب آموزش مهارت‌هاي زندگي، سعي گردد هوش هيجاني دانشجويان در زمينه مؤلفه‌هاي فوق پرورش يابد.

Title: علل بستری مجدد بيماران در بيمارستان الزهرا (س) اصفهان

http://hsr.mui.ac.ir/index.php/jhsr/article/view/191

مقدمه: برقراري تعادل بين هزينه بخشي و ارايه مراقبت با کيفيت به بيماران، يک چالش مداوم براي سيستم‌هاي مراقبت سلامت مدرن است. پذيرش مجدد نه تنها تأثیر منفي روي کيفيت زندگي بيمار و خانواده‌اش دارد، بلکه اثرات اقتصادي نامطلوبي نیز روي سيستم ارايه مراقبت سلامت دارد. بدين منظور علل مراجعات مجدد بيماران بستري به بيمارستان الزهرا تعيين گرديد. روش‌ها: اين مقاله حاصل مطالعه‌ای مقطعی بر روي پرونده‌‌هاي بيماران بستري است که در نيمه اول سال 1385 در بيمارستان الزهرا پذيرش شدند و طي یک ماه بعد از ترخيص دوباره به همان بيمارستان پذيرش داشتند. تعداد 161 پرونده مراجعه مجدد داشتند. تجزيه و تحليل داده‌ها با استفاده از نرم‌افزار آماري SPSS انجام گردید، که از آزمون‌های کاي دو و من ويتني استفاده شد. یافته‌ها: علل عمده مراجعه مجدد به بيمارستان الزهرا به شرح ذیل است: تکمیل درمان 6/36 درصد، عود بیماری 5/20 درصد، بيماري جديد 18 درصد، لغو عمل جراحي6/5 درصد، عوارض عمل جراحي 3/4 درصد، درمان ناقص 3/4 درصد،  عفونت 1/3 درصد، شکست درمان 5/2 درصد، عفونت بعد ازعمل5/1 درصد، ترخيص باميل شخصي 2/1 درصد، تشخيص نادرست 2/1 درصد. نتیجه‌گیری: عوامل متعددي در پذيرش مجدد بيماران به بيمارستان مي‌تواند دخيل باشد که عمده‌ترين آن‌ها را مي‌توان در سه حيطه عوامل مربوط به شخص بيمار، عوامل باليني وعوامل بيمارستاني نام برد.

Title: بررسی وضعیت رفتارهای پیش‌گیری ‌کننده از ابتلا و گسترش آنفلوانزای نوع (A) بر اساس تئوری انگیزش محافظت در دانش‌آموزان دختر دبیرستانی شهر اصفهان 1388

http://hsr.mui.ac.ir/index.php/jhsr/article/view/192

مقدمه: امروزه گسترش آنفلوانزای نوع (A) به طور پاندمیک بیش از گذشته، سلامت مردم جهان را تحت تأثیر قرار داده است. گفته می‌شود که اقدامات و پیش‌گیری‌های غیر دارویی در کنترل و گسترش این بیماری بسیار مؤثر است. از این رو به منظور داشتن یک مدیریت اثربخش در پاندمیک آنفلوانزای نوع (A)، شناخت عوامل مؤثر در تغییرات رفتارهای پیش‌گیری کننده قابل توجه است. این مطالعه با تعیین وضعیت رفتارهای پیش‌گیری کننده دانش‌آموزان دختر در ابتلا و گسترش آنفلوانزای نوع (A)، بر اساس تئوری انگیزش محافظت انجام گرفت.  روش‌ها: مطالعه به شیوه مقطعی با روش نمونه‌گیری چند مرحله‌ای تصادفی صورت گرفت. داده‌ها به وسیله پرسش‌نامه محقق ساخته جمع‌آوری شد که توسط 300 دانش‌آموز دختر دبیرستانی شهر اصفهان در دی‌ماه سال 88 تکمیل گردید. سؤالات پرسش‌نامه مبتنی بر تئوری انگیزش محافظت بود. آنالیز داده‌ها با استفاده از نرم‌افزار SPSS نسخه 14 و آمار توصیفی و استنباطی انجام گرفت.   یافته‌ها: میانگین سنی افراد مورد مطالعه 62/15 (1/1 = SE) سال بود. اکثر شرکت‌کنندگان در خصوص آنفلوانزای نوع (A) اطلاعات کسب کرده بودند (3/87 درصد، 300/262). یافته‌ها همبستگی مثبت و معنی‌داری را میان حساسیت درک شده، شدت درک شده و خودکارآمدی با رفتارهای پیش‌گیری‌ کننده نشان دادند (01/0 < P). ارزیابی کنار آمدن (خود کارآمدی و کارآمدی پاسخ) و ارزیابی تهدید (حساسیت درک شده و شدت درک شده) ارتباط مستقیم و معنی‌داری با رفتار داشتند و این دو با هم 11 درصد رفتار را پیش‌گویی می‌کردند. نتیجه‌گیری: نتایج این مطالعه، تهدید درک شده بالایی برای آنفلوانزای نوع (A) نشان داد. از این رو، وجود آموزش‌دهندگان سلامت عمومی و برنامه‌ریزان جهت مداخلات مبتنی بر مقابله با پاندمیک آنفلوانزای نوع (A) ضروری تلقی می‌شود. همچنین با توجه به این که مهم‌ترین منبع اطلاع‌رسانی، رسانه‌های جمعی گزارش شد. به نظر می‌رسد که سیاست‌گذاری‌های جدید باید آگاهی دانش‌آموزان را با استفاده از منابع گوناگون علمی ارتقاء دهند.

Title: بررسی تأثیر تغییر سبک زندگی با استفاده از مدل اعتقاد بهداشتی بر عوامل خطرساز قلبی درزنان یائسه شهر اهواز

http://hsr.mui.ac.ir/index.php/jhsr/article/view/207

مقدمه: هدف از این پژوهش بررسی تأثیر تغییر سبک زندگی با استفاده از مدل اعتقاد بهداشتی بر ریسک فاکتورهای قلبی- عروقی در زنان یائسه شهر اهواز بود. روش‌ها: اجرای این پژوهش کارآزمایی بالینی، از تیر ماه 1386 آغاز شد و در خرداد 1387خاتمه یافت. تعداد 67 زن یائسه با فعالیت فیزیکی کم وارد پژوهش شدند و به دو گروه مداخله (38 نفر) و گروه شاهد (29 نفر) به صورت تصادفی تقسیم شدند. در طی 6 ماه بررسی، برای گروه مداخله یا ورزش، 5 برنامه آموزشی شامل موارد زیر ترتیب داده شد: -         یک جلسه آموزش چهره به چهره در جلسه اول -         یک جلسه در پایان ماه سوم -         سه جلسه آموزشی یک ساعته با استفاده از وسایل کمک آموزشی (مانند اسلاید) در ماه اول و در پایان هر هفته. این گروه همچنین یک جزوه‌ آموزشی در مورد یائسگی، تأثیر آن بر قلب و عروق و فواید ورزش در پیش‌گیری از بیماری‌های قلبی- عروقی، که بر اساس مدل اعتقاد بهداشتی تهیه شده بود، دریافت کردند. در پایان هر ماه نیز 5 تماس تلفنی جهت یادآوری انجام ورزش با گروه مداخله برقرار می‌شد. اندازه‌گیری‌هایی شامل تغییرات در اعتقاد بهداشتی زنان، اندازه‌گیری آنتروپومتریک، فشارخون، چربی‌های خون و میزان فعالیت فیزیکی در ابتدای پژوهش و در پایان ماه ششم در دو گروه انجام شد. اطلاعات با استفاده از نرم‌افزار 15SPSS مورد بررسی قرار گرفت. از تست‌های توصیفی، تست‌های آماری شامل تست t زوجی و مستقل و تست مکرر (Repeated Measure) برای تجزیه و تحلیل داده‌ها استفاده شد. یافته‌ها: یافته‌ها بیانگر کاهش معنی‌دار در سطح چربی با دانسیته پایین (LDL به میزان 15 میلی گرم در دسی لیتر (05/0 > P)، میزان قند خون ناشتا (4/7 میلی گرم در دسی لیتر05/0 > P)، و افزایش فعالیت بدنی (به میزان 272 دقیقه در هفته، 02/0 = P) در گروه مداخله بود. تمام اجزای مدل اعتقاد بهداشتی به جز میزان آگاهی، در گروه مداخله نسبت به گروه کنترل به صورت معنی‌داری افزایش یافته بود (001/0 = P). نتیجه‌گیری: تغییر سبک زندگی با استفاده از مدل اعتقاد بهداشتی شایددر کاهش ریسک فاکتورهای بیماری‌های قلبی- عروقی در زنان بدون تحرک و کم تحرک یائسه مؤثر باشد.

Title: بررسی و مقايسه نگرش مادران مراجعه‌کننده به مراکز بهداشتی- درماني شهري اردبیل و بوکان نسبت به واکسیناسیون

http://hsr.mui.ac.ir/index.php/jhsr/article/view/199

مقدمه: ايمن‌سازي مؤثرترين و مقرون به صرفه‌ترين وسيله در دسترس براي ترقي و پيشرفت بهداشت و سلامتي كودكان است. ايمن‌سازي در شيرخواران و كودكان كه در مقابل اين گونه بيماري‌ها حساس‌تر و با عدم تكامل سيستم ايمني مواجه هستند، نقش مهم‌تري ایفا می‌کند. اين مطالعه با هدف بررسي ارزيابي‌هاي نگرشي مادران از واكسيناسيون كودكان زير يك سال در قالب دو جز از مدل اعتقاد بهداشتي (حساسيت و شدت درك شده) در سال 1388 صورت گرفت. روش‌ها: در اين مطالعه مقطعي تعداد 350 نفر مادران داراي كودك زير يك سال مراجعه‌كننده به مراكز بهداشتي درماني شهرستان اردبیل و شهرستان بوكان به روش خوشه‌اي وارد مطالعه شدند. براي جمع‌آوري داده‌ها از پرسش‌نامه‌اي مشتمل بر 20 سؤال در 3 قسمت اطلاعات دموگرافيك، ارزيابي حساسيت و شدت درك شده مادر استفاده شد. داده‌ها، با استفاده از نرم‌افزار آماری 17SPSS و آزمون‌هاي آماري كمي و كيفي مورد تجزيه تحليل قرار گرفتند. یافته‌ها: نتايج مطالعه نشان دادند كه ميانگين سني در مادران شركت‌كننده در مطالعه 26 سال است. 90 درصد مادران اثربخشي واكسن را بسیار زیاد ذکر کردند و 7 درصد مادران عقیده داشتند که واکسن‌ها بی‌اثر یا کم اثر هستند. ادراک مادران در رابطه با این جمله که «كودك من همانند ساير كودكان در مقابل بيماري‌ها حساس است» سنجیده شد که اکثریت مادران (3/58 درصد) مخالف این موضوع بودند. اکثریت مادران (9/70 درصد) موافق این جمله بودند كه «واكسن براي كودكان ضعيف مناسب‌تر است» و در این ادراک بین مادران اختلاف معنی‌داری نیز مشاهده شد (001/0 = P). نتیجه‌گیری: نگرش والدین نسبت به واکسیناسیون به طور کلی مساعد است اما مشاوره در مورد نیاز به واکسن و اطلاعات مرتبط با آن‌ها در مورد واکسن‌های در حال اجرا ضروری است. باید تلاش‌های بیشتری برای آموزش پدر و مادر در موارد منع مصرف واقعی و غیر واقعی انجام گیرد.

Title: بررسي ارتباط عوامل اقتصادی– اجتماعی و پیامدهای مرتبط در مبتلايان به ديابت نوع 2

http://hsr.mui.ac.ir/index.php/jhsr/article/view/212

مقدمه: با عنايت به نقش عوامل اقتصادي- اجتماعي در ظهور بيماري‌هاي مزمن و ضرورت و اهميت توجه به اين بعد از بيماري‌ها از منظر سياست‌گذاران و مديران کلان سازمان‌هاي بهداشتي و درماني، اين پژوهش با هدف بررسي نقش عوامل فوق بر بيماري ديابت نوع 2 و پيامدهاي آن اجرا گردید تا یافته‌هاي آن در سياست‌گذاري سيستم بهداشتي و درماني مناسب براي پيش‌گيري از وقوع اين بيماري مفيد فايده باشد. روش‌ها: اين پژوهش از نوع توصيفي- تحليلي بود. آزمودني‌هاي پژوهش 384 نفر از بيماران ديابتي مراجعه كننده به یکی از مراكز ديابت شهر اصفهان بودند که به روش نمونه‌گيري به صورت تصادفي ساده انتخاب شدند. جهت جمع‌آوري اطلاعات، از پرسش‌نامه استفاده شد و داده‌ها با استفاده از آزمون‌هاي آماري Chi Square و تست Fisher تجزيه و تحليل گردید. یافته‌ها: نتايج حاصل از پژوهش نشان داد که 1/52 درصد افراد مرد و 9/47 درصد زن بودند. بين تعداد پيامد ديابت، گروه سني، سطح تحصيلات، وضعيت اشتغال، نوع ارتباط با اعضای خانواده، دفعات ارتباط و ايجاد امنيت خاطر در بيمار ديابتي توسط خانواده، نحوه گذراندن اوقات فراغت، وضعيت سلامتي، سال‌هاي ابتلا به ديابت، استعمال دخانيات، نوع درمان، مصرف غذاي سرخ کردني و گروه درآمدي (001/0 > P)، احساس امنيت در محل زندگي و ارتباط در محل زندگي (002/0 > P) و نیز مصرف روزانه گروه سبزيجات (02/0 > P) از نظر آماري ارتباط معني‌داري مشاهده شد. نتیجه‌گیری: به نظر مي‌رسد به کارگيري استراتژي‌هاي کمکي و منابع حمايتي در افراد مبتلا به بیماری‌های مزمن به خصوص دیابت، مسأله‌اي حياتي است. استراتژي‌هاي توانمند سازي باعث ايجاد تغييرات فاحش در افراد مي‌شود تا انتظارات مثبت، اميد، عزت نفس و اطمينان خود را افزايش دهند؛ اين همان راهکاري است که لازم است در بيماران ديابتي به منظور کنترل هر چه بهتر بيماري و انواع مختلف عوارض آن به کار گرفته شود.
